# High-fat diet and palmitic acid amplify airway type 2 inflammation

**DOI:** 10.3389/falgy.2023.1193480

**Published:** 2023-05-23

**Authors:** Kris Genelyn Dimasuay, Bruce Berg, Niccolette Schaunaman, Fernando Holguin, Daniel Winnica, Hong Wei Chu

**Affiliations:** ^1^ Department of Medicine, National Jewish Health, Denver, CO, United States; ^2^ Department of Medicine, University of Colorado Anschutz Medical Campus, Aurora, CO, United States

**Keywords:** obesity, asthma, palmitic acid, IL-13, type 2 inflammation

## Abstract

**Introduction:**

Metabolic dysfunction such as elevated levels of saturated fatty acids (SFA) may play a role in obese asthma, but its contribution to airway inflammation remains unclear. We sought to determine the role of high-fat diet (HFD) and palmitic acid (PA), a major form of SFA, in regulating type 2 inflammation.

**Methods:**

Airway samples from asthma patients with or without obesity, mouse models and human airway epithelial cell culture were utilized to test if SFA amplify type 2 inflammation.

**Results:**

Asthma patients with obesity had higher levels of airway PA than asthma patients without obesity. HFD increased the levels of PA in mice, and subsequently enhanced IL-13-induced airway eosinophilic inflammation. PA treatment amplified airway eosinophilic inflammation in mice that were previously exposed to IL-13 or house dust mite. IL-13 alone or in combination with PA increased dipeptidyl peptidase 4 (DPP4) release (soluble DPP4) and/or activity in mouse airways and human airway epithelial cells. Inhibition of DPP4 activity by linagliptin in mice pre-exposed to IL-13 or both IL-13 and PA increased airway eosinophilic and neutrophilic inflammation.

**Discussion:**

Our results demonstrated the exaggerating effect of obesity or PA on airway type 2 inflammation. Up-regulation of soluble DPP4 by IL-13 and/or PA may serve as a mechanism to prevent excessive type 2 inflammation. Soluble DPP4 may have the therapeutic potential in asthma patients with obesity who have an endotype with mixed airway eosinophilic and neutrophilic inflammation.

## Introduction

Prevalence of obesity is increasing globally with at least 500 million adults predicted to be obese by 2030 (1). Obesity is one of the major risk factors for the development of asthma. Patients with obesity have more severe asthma than lean subjects, and have a unique phenotype characterized by increased exacerbations, greater severity, poor quality of life, and poor responses to conventional asthma therapy (2–4). However, the mechanisms by which obesity affects asthma pathogenesis and severity remain poorly understood.

Studies have shown that obesity is associated with type 2 cytokine-high inflammation. Several groups of investigators have demonstrated enhanced recruitment of eosinophils in the lungs of asthma patients with obesity compared to lean asthma patients (5–7). Additionally, body mass index (BMI) was shown to positively correlate with type 2 cytokines IL-5 and IL-13 in sputum samples of asthmatic individuals (8). These studies suggest that although obese asthma presents a non-type 2 phenotype, there is still a large subgroup of asthma patients with obesity presenting a type 2 inflammation-high endotype (9).

One of the major causes of obesity is the long-term consumption of high-fat and high-caloric diets (10). High-fat diets were shown to increase the levels of circulating saturated fatty acids (SFA) that can activate inflammatory signaling such as ROS, NF-κB, MAPK, and modulate innate immune responses (10, 11). In individuals with obesity, levels of SFA increased in both serum and plasma samples (12, 13). Therefore, increased levels of SFA may potentially contribute to increased inflammatory signaling seen in patients with obese asthma. In obese asthma, the role of SFA such as palmitic acid (PA) in the eosinophilic inflammatory response is still unknown. Here, we hypothesized that SFA such as PA is increased in asthma patients with obesity, which exacerbates type 2 airway inflammation in part by modulating IL-13-mediated release of soluble dipeptidyl peptidase 4 (DPP4). DPP4 or CD26 is a homodimer transmembrane glycoprotein with a short cytoplasmic tail, a transmembrane region and a long extracellular domain (14). DPP4 is expressed by many types of cells including epithelial cells, endothelial cells and immune cells such as myeloid cells, T cells and B cells. DDP4 exerts its functions through its enzymatic activity to regulate the levels or activity of proteins or peptides. DPP4 also interacts with its binding partners to participate in cell adhesion, tissue remodeling and others. DPP4 extracellular domain can shed from the cell surface by proteases such as matrix metalloproteinases (MMPs) (15) to generate soluble DPP4 that essentially maintains the function of full-length DPP4. A previous study showed that DDP4 expression was increased in asthmatic airway epithelium, and IL-13 induced the expression of DPP4 (16). Moreover, levels of DPP4 in the serum samples of asthma patients have been proposed as a type 2 inflammation marker and may predict the therapeutic efficacy of an IL-13 neutralizing antibody in a clinical trial (17). The role of DDP4 in airway eosinophilic inflammation remains controversial. While some studies suggested DPP4 as a mediator to promote eosinophilic inflammation (18), others found the opposite or no effect of DPP4 on eosinophilic inflammation (19–21). Our current study utilized airway samples from asthmatic subjects, airway epithelial cell culture, and animal models to determine the regulation of DPP4 by airway type 2 inflammation in an obese condition such as high-fat diet or PA, and its role in type 2 inflammation.

## Materials and methods

### Bronchoalveolar lavage (BAL) fluid from human subjects

Cell-free BAL fluid (BALF) samples were obtained from asthmatic patients who are either lean (BMI < 25) or obese (BMI > 30) from the Human Cell Core at National Jewish Health. DPP4 activity and protein levels were measured in BALF. For free fatty acid analysis, BALF samples were sent to the Nutrition Obesity Research Center (NORC) Lipidomics Core Laboratory at the University of Colorado Anschutz Medical Campus for measuring free fatty acids using gas chromatography/mass spectrometry (GC/MS) Analysis. The human study was approved by National Jewish Health Institutional Review Board (IRB). All patients received spirometry tests. Asthma diagnosis was based on clinical history and medication use of β2 agonists and corticosteroids. Patients with current or past smoking were excluded from the study. Table 1 showed the clinical information.

**Table 1:** Clinical characteristics of asthma patients.

	Non-obese asthma (*n* = 8)	Obese asthma (*n* = 8)	*p* value
Age, years	36.1 ± 21.4	56.3 ± 13.9	0.01
Gender	3M/5F	4M/4F	>0.05
FEV1 (% predicted)	97.6 ± 8.2	69.2 ± 6.7	<0.01
BMI	21.3 ± 2.1	35.8 ± 5.5	<0.01

Asthma patients with or without obesity were on inhaled corticosteroids.

### Mouse model of high-fat diet (HFD) administration and IL-13 treatment

Mice were purchased from The Jackson Laboratory (Bar Harbor, Maine, USA) and maintained at the Biological Resources Center of National Jewish Health. All animal procedures were approved following the guidelines of our Institutional Animal Care and Use Committee (protocol #AS2792-03-20). Both male and female mice were used in all the experiments.

To determine the effect of obesity on the type 2 inflammatory response induced by IL-13, we established a mouse model of obesity induced by HFD administration followed by IL-13 treatment. Wild-type C57BL/6 mice of 5-weeks old were fed with either normal chow as a control (2019S, Teklad, Envigo) or with HFD (TD.06414, Teklad, Envigo) for 16 weeks. The HFD provided 60% energy in the form of fat with an approximate fatty acid profile (% of total fat) of 36% saturated, 41% monounsaturated and 23% polyunsaturated fatty acid. The normal chow contained 1.2% saturated, 1.7% monounsaturated and 4.4% polyunsaturated fatty acid. Body weight was measured every week. On week 15, blood was collected from the submandibular vein to measure glucose levels using a glucometer (one Touch Ultra-2). On the final week (week 16) of HFD treatment, mice were inoculated intranasally with IL-13 as we previously reported (22). Briefly, recombinant mouse IL-13 (Peprotech, Rocky Hill, New Jersey, USA) at 250 ng/mouse prepared in 0.1% bovine serum albumin (BSA) or 0.1% BSA (control) were intranasally administered once daily for three consecutive days. After 24 h of the last IL-13 treatment, mice were sacrificed to obtain BALF and lung tissue. BAL cells were used for leukocyte counts. Briefly, cytospins of BAL cells were stained with Diff-Quick Kit (IMEB INC., San Marcos, CA, USA), and cell differentials were determined as percentages of 500 counted cells (23). Cell-free BALF was used to measure proinflammatory mediators using ELISA. Additionally, cell-free BALF was analyzed for free fatty acids using GC/MS Analysis.

### Mouse model of lung IL-13 and palmitic acid (PA) treatment

To determine the effect of PA on established type 2 inflammation, we performed a mouse model of IL-13 treatment, followed by PA treatment. Wild-type BALB/c mice of 9–12 weeks old were intranasally treated with IL-13 as described above. Four hours before the IL-13 challenges on days 2 and 3 of IL-13 treatment, mice were administered with 100 µM PA or 0.1% fatty acid-free BSA-PBS (control) via oropharyngeal inoculation. PA was prepared by making a 10 mM stock solution in 100% ethanol and conjugated with 0.1% fatty acid-free BSA as described (24, 25). Mice were sacrificed after 24 h of the last IL-13 and/or PA for measuring lung inflammation. In a separate experiment, in order to test the role of DPP4 in airway eosinophilic inflammation, a DPP4 inhibitor linagliptin (500 µM in 50 µl saline, MedChemExpress LLC, Monmouth Junction, NJ) or saline (control) was administered oropharyngeally to mice after 24 h of the last IL-13 and/or PA. Mice were sacrificed after 24 h of linagliptin treatment. The dose of linagliptin was chosen based on our preliminary study where linagliptin at 500 µM, but not 100 µM, was shown to reduce airway eosinophil levels in IL-13-treated mice.

### Mouse model of house dust mite (HDM) challenges and PA treatment

To determine the effect of PA on established airway allergic inflammation, we performed a mouse model of HDM challenges, followed by PA treatment. Wild-type BALB/c mice of 9–12 weeks old were intranasally sensitized with HDM extracts at 10 µg/mouse or 50 µl PBS (control) on days 0 and 7. Mice were then challenged once a day for 3 consecutive days on days 14, 15 and 16 with 10 µg HDM or 50 µl PBS via intranasal inoculation. Two days after the last HDM challenge, mice were administered with 100 µM PA or 0.1% fatty acid-free BSA-PBS (control) via oropharyngeal inoculation as described above. Mice were sacrificed after 24 h of PA treatment for measuring lung inflammation.

### Primary human tracheobronchial epithelial (HTBE) cell culture and treatments

Detailed method on isolation of primary HTBE cells from normal healthy donors were described in our previous publication (26). Isolated HTBE cells were expanded on collagen-coated 100 mm dishes containing BronchiaLife medium with supplements (Lifeline Cell Technology, Frederick, MD, USA). Air–liquid interface (ALI) culture was performed by seeding the cells onto collagen-coated 12-well transwell plates (Transwell 2460, Corning Incorporated, Corning, New York, USA) with PneumaCult-ALI medium (StemCell, Vancouver, British Columbia, Canada). After 7 days of submerged culture, cells were shifted to ALI for the next 21 days to induce mucociliary differentiation. Cells were stimulated with 10 ng/ml recombinant human IL-13 (R&D Systems, Minneapolis, Minnesota, USA) for three consecutive days starting on day 21 of ALI. On days 22 and 23, cells were treated with 100 µM PA conjugated with 0.1% fatty acid-free BSA or 0.1% fatty acid-free BSA as a control. Cells, apical and basolateral supernatants were harvested on day 24 (24 h after the last IL-13/PA treatment). Cells were lysed with RIPA buffer for western blot analysis. Supernatants were used for the DPP4 activity assay and ELISA.

### Western blot

Cell supernatants (equal volume for all the samples) were prepared and analyzed as previously described (26, 27). Blots were probed with an antibody against DPP4 (1:1,000, Proteintech) followed by horseradish peroxidase-conjugated secondary IgG (1:3,000; EMD Millipore, Burlington, Massachusetts, USA).

### ELISA

Human eotaxin-3, human DPP4 and mouse eotaxin-2 were measured using Duoset ELISA kits (R&D Systems) according to manufacturer's instructions. ELISA results from cell culture supernatants were normalized to protein content of cell lysate.

### DPP4 enzymatic activity

DPP4 activity in BALF and cell culture supernatants was measured using an assay kit purchased from Sigma (MAK088) according to manufacturer's protocol. Initial measurement was taken after 5 min of incubation at 37°C while the final measurement was taken after 60 min. DPP4 activity was reported as μUnit/ml, which is equivalent to pmole/min/ml.

### Oil Red O staining in mouse liver tissue

To examine lipid deposition in liver tissue of mice fed with HFD, Oil Red O staining was performed (28). Briefly, frozen liver tissue sections cut at 5 µm thickness were fixed in 4% paraformaldehyde for 20 min at room temperature, followed by incubation with 100% propylene glycol for 5 min and then with 0.5% Oil Red O solution for 20 min at room temperature. The slides were counterstained with hematoxylin solution for 1 min and covered with an aqueous mounting medium.

### Statistical analyses

Data were analyzed and graphs were designed using Prism 9 software (Graph Pad). For parametric data, two-group comparisons were made using the Student's *t*-test, and multiple comparisons were performed using one-way ANOVA with Holm-Sidak's *post hoc* test. Correlation was assessed using the Pearson coefficient. For non-parametric data, two-group comparisons were done using the Mann–Whitney test, and multiple comparisons were made using the Kruskal–Wallis test. A *p* value <0.05 was considered statistically significant.

## Results

### Increased PA and soluble DPP4 levels in BAL fluid of human asthma patients with obesity

**Figure 1:**
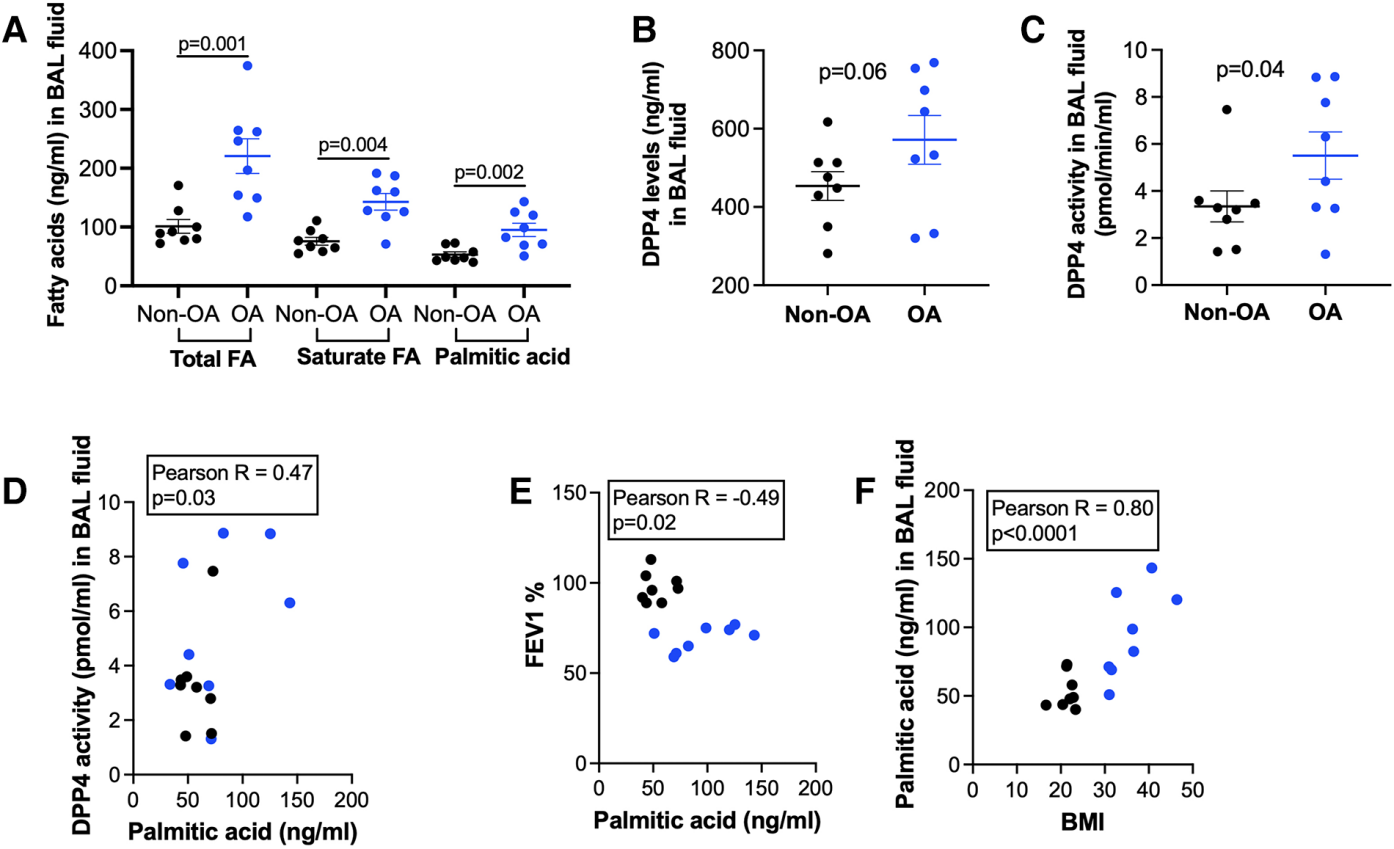
Levels of fatty acids and DPP4 in bronchoalveolar lavage (BAL) fluid of patients with non-obese asthma (non-OA, *n* = 8) and with obese asthma (OA, *n* = 8). (**A**) Levels of total and saturated fatty acids (FA) and palmitic acid; (**B**) DPP4 levels in non-OA and OA patients; (**C**) DPP4 activity levels in non-OA and OA patients. The horizontal bars indicate means ± SEM. (**D,E**) Correlations of palmitic acid levels with DPP4 activity in BAL fluid and forced expiratory volume in 1 s (FEV1) % predicted in non-OA and OA patients; (**F**) Correlations of body mass index (BMI) with palmitic acid levels in BAL fluid of non-OA and OA patients. Black and blue dots indicate non-OA and OA groups, respectively.

BAL fluid samples were available from patients with non-obese and obese asthma (Table 1 showing the demographic data). As shown in Figure 1A, patients with obese asthma had significantly higher levels of total fatty acids (FA), saturated FA and PA in BAL fluid than patients with non-obese asthma. Levels of soluble DPP4 protein and DPP4 activity were also higher in patients with obese asthma (Figures 1B,C). PA positively correlated with DPP4 activity (Figure 1D), but negatively correlated with forced expiratory volume in 1 s (FEV1) % predicted, an indicator of airway obstruction (Figure 1E). BMI strongly and positively correlated with the levels of PA (Figure 1F).

### High-fat diet amplified type 2 inflammation in IL-13-treated mice

**Figure 2:**
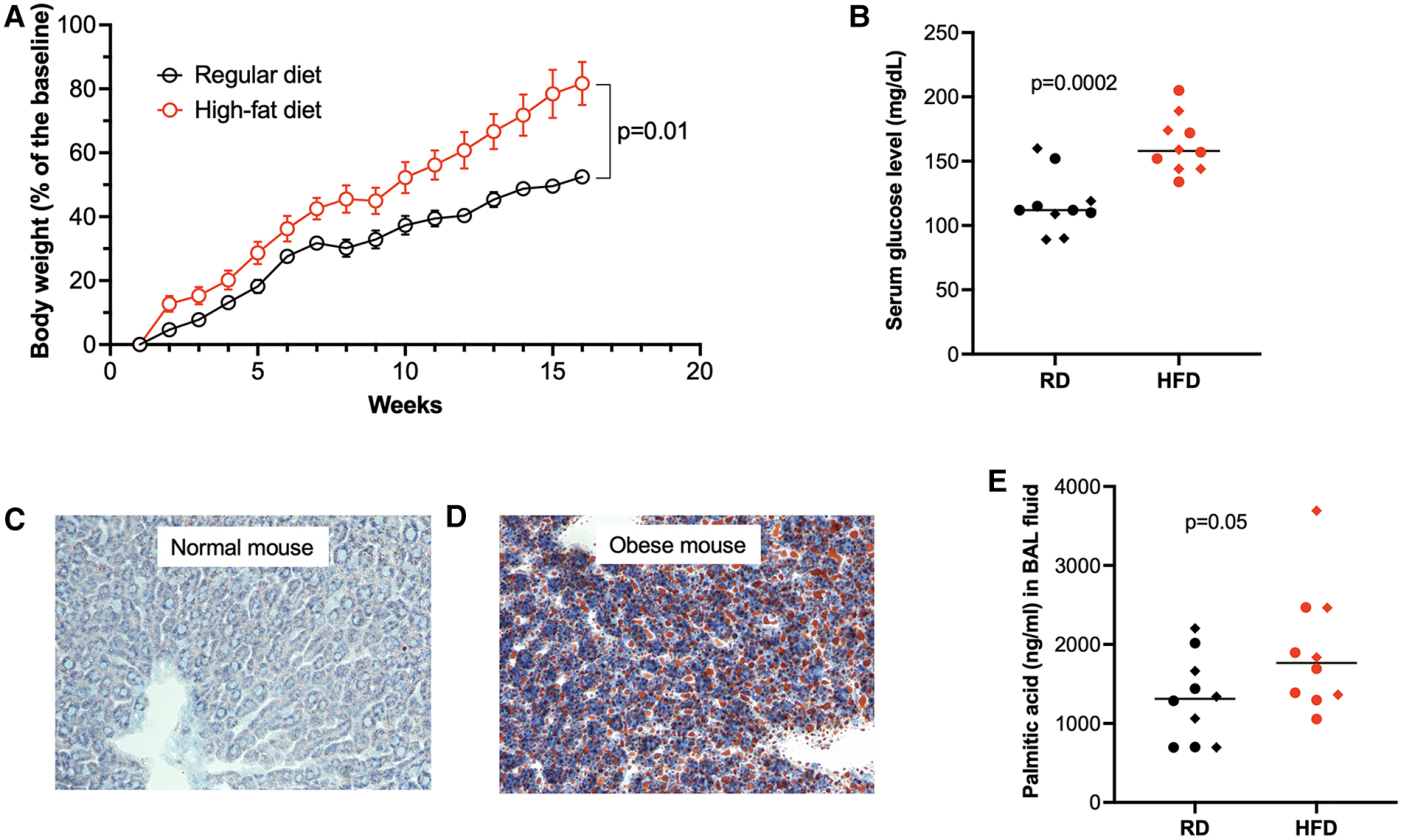
High-fat diet (HFD) increased body weight (**A**), serum glucose levels (**B**), lipid deposition in the liver (**C,D**, Oil Red O staining), and palmitic acid in bronchoalveolar lavage (BAL) fluid (**E**). RD, regular diet. Diamonds indicate female mice, and full circles indicate male mice in **B,E**. The horizontal bars indicate medians.

To investigate the role of obesity in type 2 inflammation, mice were first fed with high-fat diet (HFD) or regular diet (normal control) for 15 weeks. In week 16, mice receiving HFD or regular diet were intranasally inoculated with IL-13 to induce type 2 inflammation. Mice fed with HFD (obese mice) versus mice fed with regular diet gained about 40% of more body weight (Figure 2A), and had significantly higher levels of glucose in the serum (Figure 2B). Obese mice demonstrated more lipid deposition in the liver (Figures 2C,D). Importantly, PA levels in BAL fluid (Figure 2E) of obese mice were higher than those in BAL fluid of mice with regular diet.

**Figure 3:**
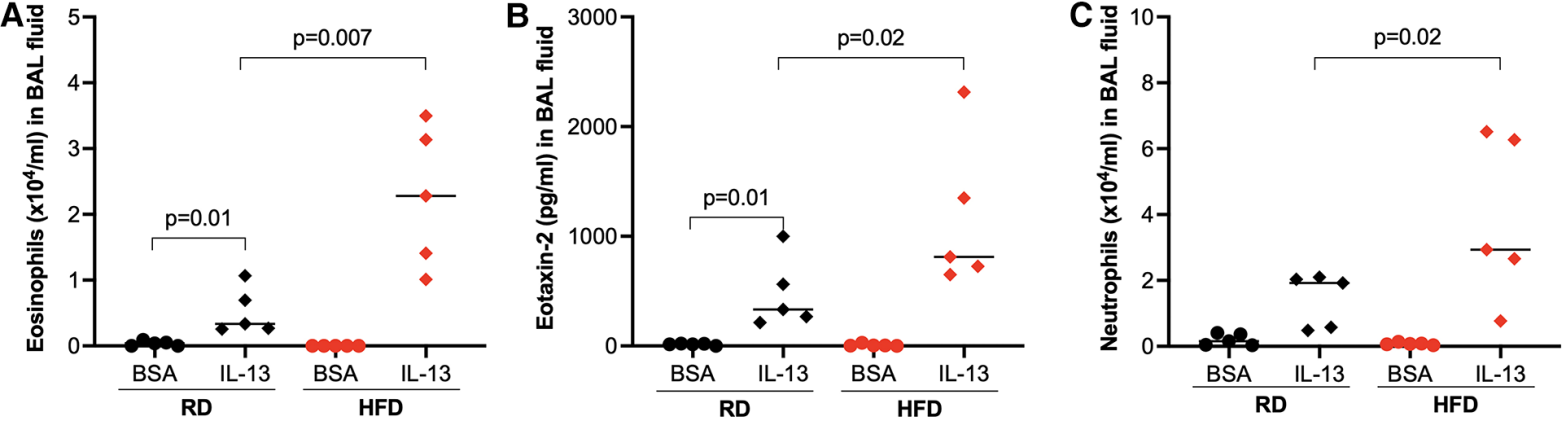
High-fat diet (HFD) increased eosinophils (**A**), eotaxin-2 (**B**), and neutrophils (**C**) in bronchoalveolar lavage (BAL) fluid of IL-13-treated mice. Mice were fed with regular diet (RD) or HFD for 16 weeks, followed by 3 consecutive days of intranasal IL-13 treatments. BAL was obtained after 24 h of the last IL-13 treatment. *N* = 5 mice/group. The horizontal bars indicate medians.

Following IL-13 treatments, the number of eosinophils (Figure 3A) and levels of eotaxin-2 (an eosinophilic chemoattractant) (Figure 3B) in BAL fluid were significantly increased in HFD-fed obese mice compared with mice fed with regular diet. Additionally, HFD also increased the levels of neutrophils in IL-13-treated mice (Figure 3C). Our data suggests that obesity may enhance IL-13-mediated airway type 2 inflammation as well as neutrophilic inflammation.

### PA exaggerated airway type 2 inflammation in mice pre-exposed to IL-13 or house dust mire (HDM)

**Figure 4:**
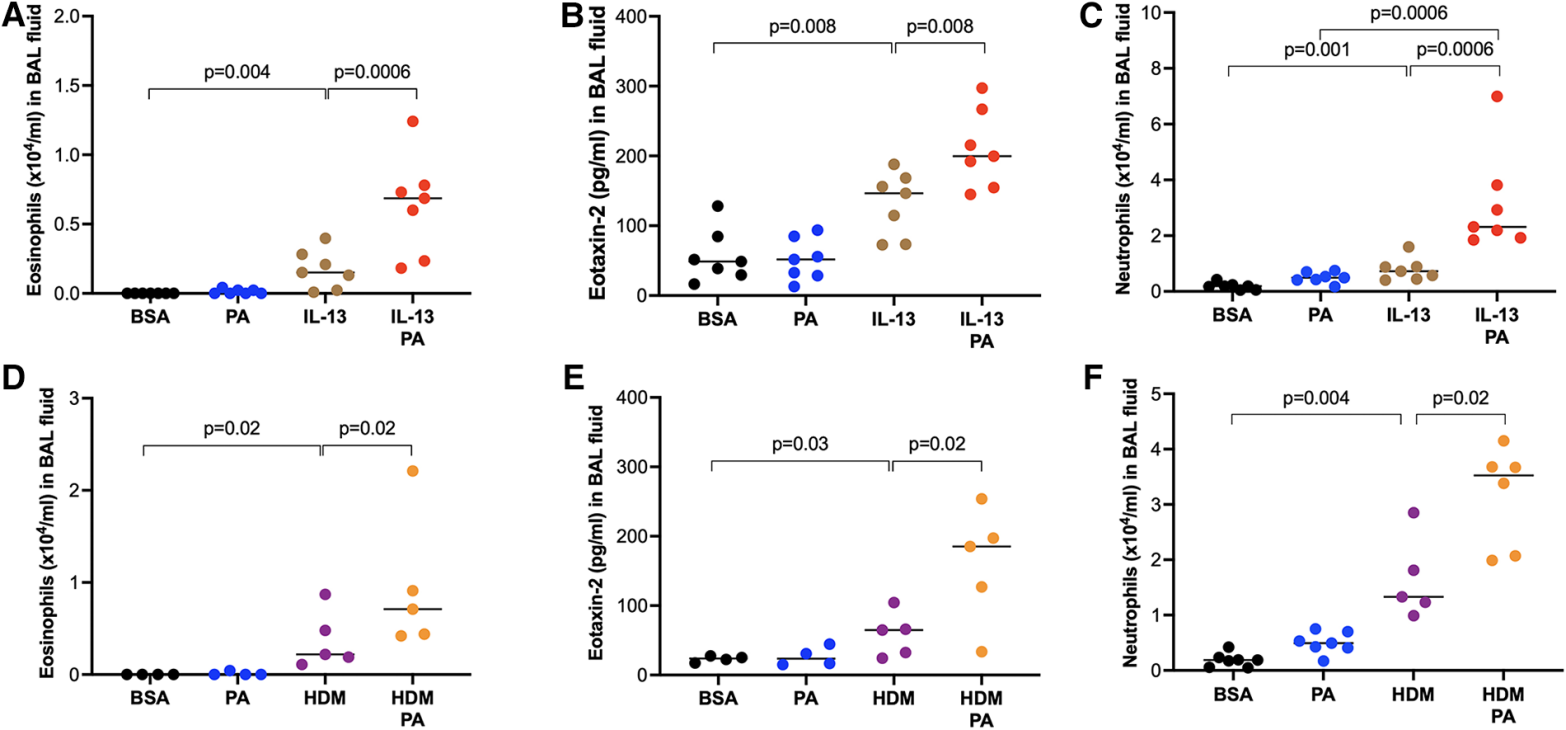
Palmitic acid (PA) exacerbated airway inflammation in IL-13 or house dust mite (HDM)-challenged mice. Wild-type BALB/c mice were exposed to IL-13 or HDM, and then treated with PA as described in the Materials and methods section. (**A–C**) Eosinophil, eotaxin-2 and neutrophil levels in bronchoalveolar lavage (BAL) fluid of mice treated with IL-13 and PA. Bovine serum albumin (BSA) and PA alone served as controls. (**D–F**) Eosinophil, eotaxin-2 and neutrophil levels in bronchoalveolar lavage (BAL) fluid of mice treated with HDM and PA. *N* = 4–7 mice/group. The horizontal bars indicate medians.

Having demonstrated the role of obesity in modulating the subsequent type 2 inflammation, we next determined if PA promotes pre-existing type 2 inflammation induced by IL-13 or HDM. Mice challenged with IL-13 and then treated with PA had significantly higher levels of eosinophils (Figure 4A) and eotaxin-2 (Figure 4B) in BAL fluid than mice treated with IL-13 alone. Similarly, HDM-challenged mice treated later with PA showed higher levels of eosinophils (Figure 4D) and eotaxin-2 (Figure 4E) in BAL fluid. Neutrophil levels were also increased by PA in IL-13-treated (Figure 4C) or HDM-treated (Figure 4F) mice.

### PA amplified IL-13-induced release of eotaxin-3 and soluble DPP4 in primary human airway epithelial cells cultured at air-liquid interface

**Figure 5:**
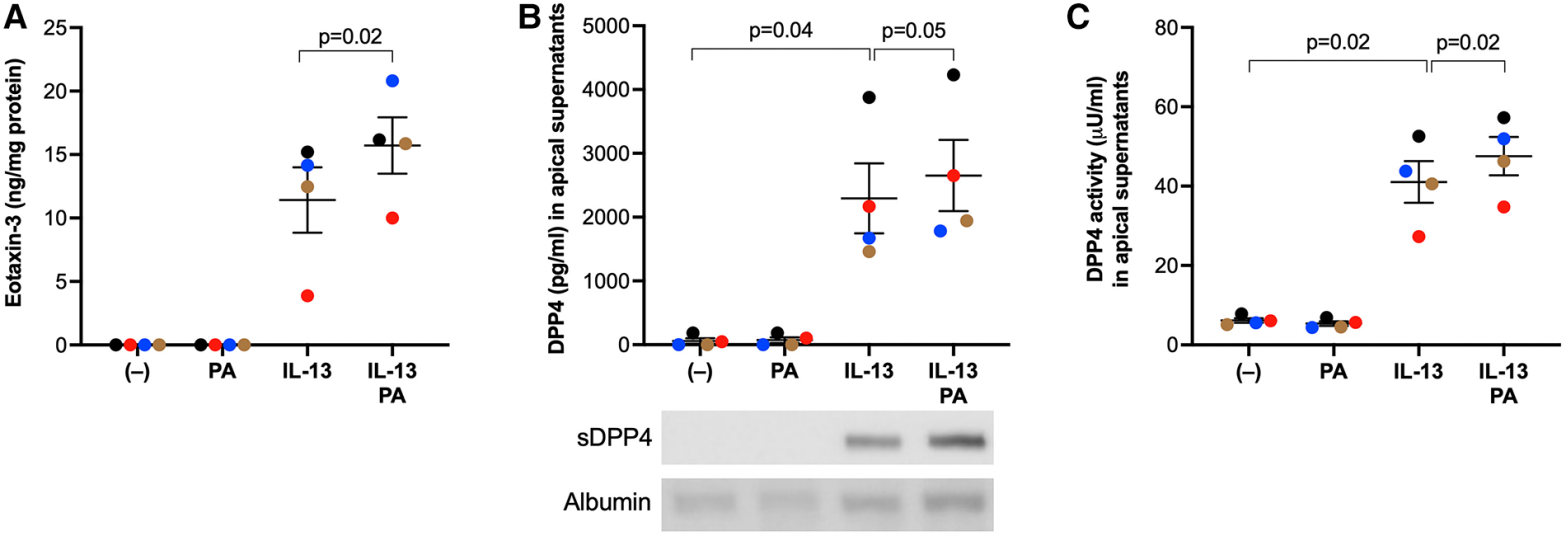
IL-13 and palmitic acid (PA) increased eotaxin-3 and DPP4 levels in cultured human primary airway epithelial cells. Normal human tracheobronchial epithelial cells were cultured at air-liquid interface, and treated with IL-13 and PA as described in the Materials and methods section. (**A**) Levels of eotaxin-3 measured in basolateral supernatants of cultured airway epithelial cells; (**B**) Levels of DPP4 protein measured in apical supernatants of cultured airway epithelial cells using ELISA and western blot (25 µl/sample). sDDP4 = soluble DPP4. Albumin revealed by Amido black staining served as a loading control. (**C**) Levels of DPP4 activity in apical supernatants of cultured airway epithelial cells. *N* = 4 donors. The horizontal bars indicate means ± SEM.

To determine if human airway epithelial cells responded to PA and IL-13 in a similar manner to mice, we measured eotaxin-3 and soluble DPP4 in cultured human primary airway epithelial cells. Similar to what was found in the mouse model, PA stimulation in IL-13 pre-exposed cells increased the production of eosinophil chemoattractant eotaxin-3 (Figure 5A) coupled with more soluble (Figure 5B) and active (Figure 5C) DPP4.

### Inhibition of DPP4 activity enhanced eosinophil and eotaxin-2 levels in BAL fluid of mice exposed to IL-13

**Figure 6:**
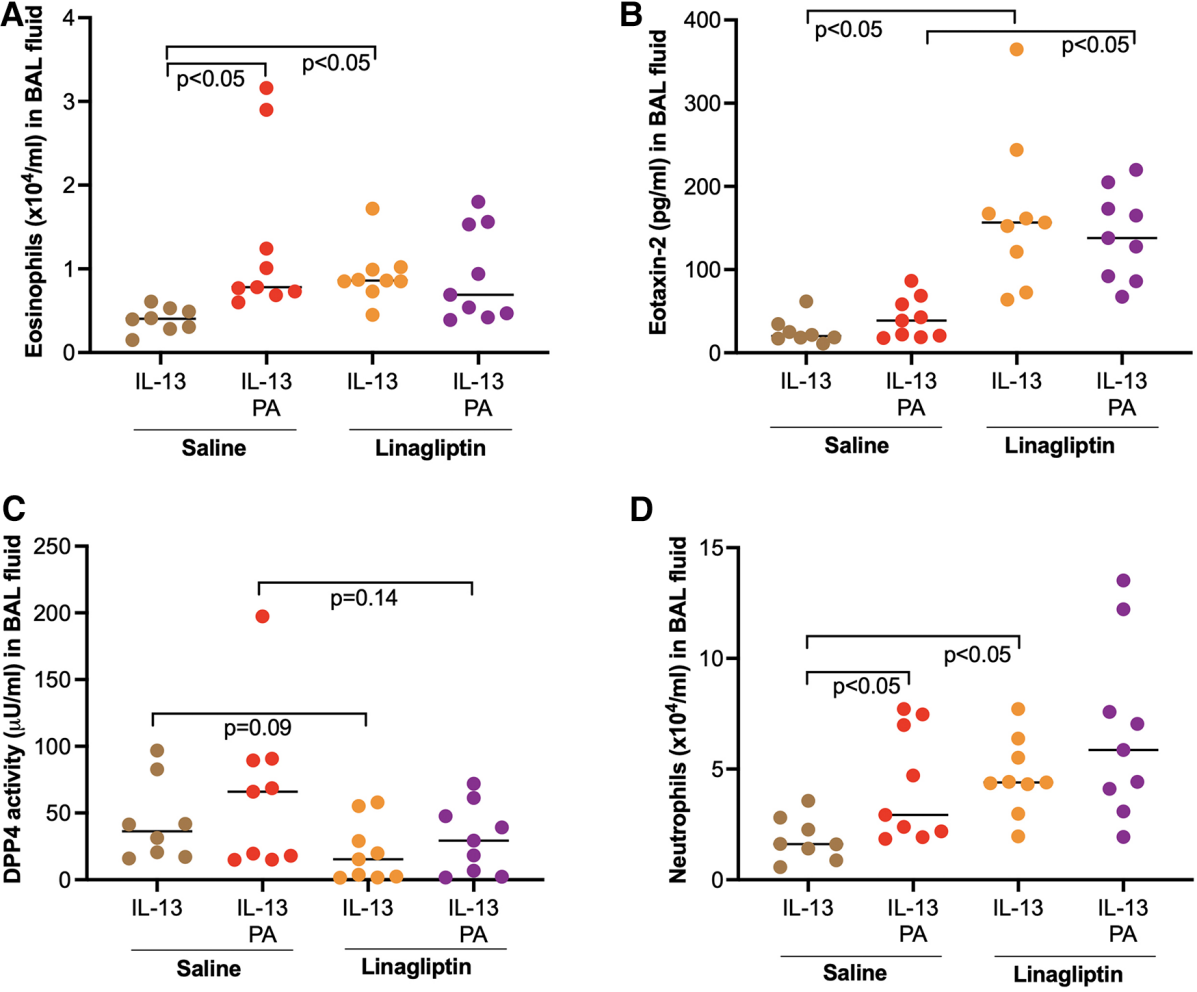
Effect of a DPP4 inhibitor linagliptin on airway inflammation in mice exposed to IL-13 and palmitic acid (PA). (**A,B**) Levels of eosinophils and eosinophil chemoattractant eotaxin-2 in bronchoalveolar lavage (BAL) fluid; (**C**) Levels of DPP4 activity in BAL fluid; (**D**) Levels of neutrophils in BAL fluid. *N* = 8–9 mice/group. The horizontal bars indicate medians.

Our data above showed the amplifying effect of PA on IL-13-iduced airway eosinophilic inflammation. When linagliptin, a selective inhibitor of DPP4 activity, was administered intranasally to mice treated with IL-13 alone or combination of PA and IL-13, linagliptin significantly increased the levels of eosinophils and eosinophil chemoattract eotaxin-2 in BAL fluid of mice treated with IL-13 alone or combination of PA and IL-13 (Figures 6A,B). Interestingly, eosinophil levels were not altered by linagliptin in mice treated with both IL-13 and PA. PA trended to increase DPP4 activity in IL-13-treated mice (Figure 6C). DDP4 activity was moderately reduced by linagliptin (Figure 6C). Additionally, neutrophil levels in BAL fluid were significantly increased by linagliptin treatment in mice exposed to IL-13 (Figure 6D).

## Discussion

Although saturated fatty acids have been reported to be associated with obesity and obese asthma, their role in modulating airway inflammation has not been well understood. Our current study has revealed that high-fat diet and palmitic acid amplified airway type 2 inflammation that was established before or after high-fat diet and palmitic acid were administered. We also found that enhanced release of soluble DDP4 mediated by palmitic acid in a type 2 inflammation-high environment may serve as a mechanism to prevent excessive airway eosinophilic inflammation.

Heterogeneity of airway inflammation has been reported in obese asthma. While some patients demonstrate an endotype of predominant airway neutrophilic inflammation, others show more eosinophils in the blood and airways (29–31). The mechanisms behind the heterogenous nature of airway inflammation in obese asthma remain unclear. Our human data revealed that saturated fatty acids (e.g., palmitic acid) are increased in airways of patients with obese asthma. Palmitic acid has been shown to activate various signaling pathways (e.g., TLR4) in macrophages to induce the proinflammatory response (32). How palmitic acid signaling interacts with or regulates type 2 inflammation has not been well studied. A previous study in mice (33) demonstrated that mice exposed to HFD or palmitic acid followed by HDM challenges increased airway neutrophilic inflammation, but not eosinophilic inflammation. In our current study, we treated HFD-fed mice with IL-13, but not HDM. Like the previous study, we found that HFD increased neutrophil levels in IL-13-treated mice. However, we also found higher levels of airway eosinophils in HFD- and IL-13-treated mice. Moreover, in mice pretreated with IL-13 or HDM, palmitic acid increased lung eosinophilic inflammation as well as neutrophilic inflammation. Thus, our study has extended the previous study by showing increased lung neutrophilic inflammation by PA after the allergic or type 2 inflammation has been established. Our novel finding that palmitic acid or HFD increases both eosinophilic and neutrophilic inflammation may potentially explain why obese asthma is difficult to manage or treat. Previous studies suggest that asthma patients with an endotype of mixed-granulocytic inflammation (increase in both eosinophils and neutrophils) have more severe disease such as airway obstruction, frequent asthma exacerbations and health care utilization than patients with either eosinophilic or neutrophilic inflammation (34, 35). Targeting both airway eosinophilic and neutrophilic inflammation in a subset of asthma patients with obesity could be an effective approach to reduce the severity of obese asthma.

The mechanisms by which HFD or palmitic acid enhances eosinophilic inflammation has not been well understood. In our study, we explored how HFD or palmitic acid may regulate the expression or activity of DDP4 induced by type 2 cytokine IL-13. Our results supported previous findings that IL-13 increased DPP4 production (16). Importantly, we demonstrated the ability of HFD or palmitic acid to enhance the release of soluble DPP4. Whether soluble DDP4 regulates eosinophilic inflammation has not been clear. By using a selective DPP4 inhibitor, we found that inhibition of soluble DPP4 activity amplified eosinophilic inflammation induced by IL-13. Our data for the first time suggests that soluble DPP4 may serve as a protective mechanism against excessive airway eosinophilic inflammation. Although the exact mechanisms involved in the protective role of DPP4 in eosinophil recruitment are still unclear, we found that the DPP4 inhibitor was able to increase the levels of eosinophil chemoattractant eostaxin-2 in mouse airways. Our findings supported the study by Forssmann et al. who demonstrated that DPP4 deficient rats had increased eosinophil recruitment after intravenous or intradermal administration of eotaxin-1 (CCL11) (19). Further, the authors suggest that the truncation of eotaxin-1 at the N-terminus by DPP4 may be responsible for the inhibitory effect of DPP4 on eosinophilic inflammation. In a preliminary study, we incubated recombinant human DPP4 with human recombinant eotaxin-3 protein, but did not found the truncation or degradation of eotaxn-3 by DPP4. It is unclear whether DPP4 may specifically truncate eotaxin-1. The mechanisms by which soluble DPP4 inhibits eosinophilic inflammation warrant further studies.

There are several limitations in our current study. First, in our retrospective clinical cohort of obese asthma, eosinophil data in BAL fluid was not available, which prevented us from correlating palmitic acid and DPP4 levels with airway eosinophilic inflammation in human subjects. Second, the role of DPP4 inhibitor linagliptin was only examined in the mouse model of IL-13 and PA treatment, but not in our other mouse models described in this study. Although we expect similar effects of DPP4 inhibition on airway eosinophilic inflammation in different mouse models, future experiments will be needed to confirm this hypothesis. We found that in mice treated with both IL-13 and PA, the effect of linagliptin on airway eosinophil and eotaxin-2 levels was not consistent. While linagliptin significantly increased the eotaxin-2 levels, it did not do so for airway eosinophil count. While we do not know the exact mechanisms behind this observation, we propose that airway eosinophil levels may be regulated by multiple mechanisms including eosinophil chemoattractant eotaxin-2 and factors that regulate eosinophil survival or apoptosis such as IL-5 and hydrogen peroxide (36). Furthermore, delivery of one dose of linagliptin moderately, but not significantly reduced the activity of DDP4. Although we observed a significant role of DPP4 in IL-13-induced airway eosinophilic inflammation, the contribution of DPP4 to airway eosinophilic inflammation mediated by IL-13 and PA remains unclear. Future studies can be designed to investigate the role of multiple doses of DPP4 in airway inflammation induced by IL-13 and PA or by HFD and IL-13 or HDM. Third, our current study used two different strains of mice (C57BL/6 and BALB/c) to study the impact of HFD or PA on airway inflammation in order to mimic different genetic background in human subjects. However, we did not directly compare the genetic background effect in our three mouse models. Nonetheless, previous studies suggest that C57BL/6 mice are highly susceptible to HFD-induced obesity (37). On the other hand, following HDM challenges, BALB/c have been shown to generate more severe airway type 2 inflammation than the C57BL/6 mice (38, 39). Thus, depending on the research questions, both C57BL/6 and BALB/c mice may be appropriate in the study of obese asthma.

In summary, our research findings suggest that metabolic dysfunction related to obese conditions may drive an endotype of mixed airway eosinophilic and neutrophilic inflammation, making the asthma control more challenging. Soluble DPP4 may be a potential therapeutic target for obese asthma.

## Data Availability

The original contributions presented in the study are included in the article, further inquiries can be directed to the corresponding author.
